# Outcomes of First Subsequent Taxane Therapy in Patients with Metastatic Castration-Resistant Prostate Cancer Who Previously Received Docetaxel Intensification for Metastatic Castration-Sensitive Prostate Cancer

**DOI:** 10.3390/curroncol31090375

**Published:** 2024-08-29

**Authors:** Gabrielle Robin, Naveen S. Basappa, Scott North, Sunita Ghosh, Michael Kolinsky

**Affiliations:** 1 Department of Medicine, University of Alberta, 11230-83 Ave NW, Edmonton, AB T6G 2B7, Canada; 2 Department of Medical Oncology, University of Alberta, Edmonton, AB T6G 1Z2, Canada; 3 Cross Cancer Institute, Edmonton, AB T6G 1Z2, Canada; 4 Henry Ford Hospital, One Ford Place, Detroit, MI 48202, USA

**Keywords:** FST, docetaxel intensification, taxanes, prostate cancer, mCSPC, mCRPC

## Abstract

Background: The management of advanced prostate cancer continues to evolve rapidly, particularly with the earlier use of survival-prolonging therapies in metastatic castration-sensitive prostate cancer (mCSPC). Though approved prior to the use of intensification therapy in mCSPC, taxane-based chemotherapies remain a relevant option for patients with metastatic castration-resistant prostate cancer (mCRPC). However, there is little evidence determining the outcomes of taxane chemotherapies as the first subsequent taxane (FST) in mCRPC pts who received docetaxel intensification (DI) in mCSPC. The purpose of this study is to compare outcomes between the survival-prolonging taxanes, docetaxel and cabazitaxel as FST after DI. Methods: New patient consults seen at the Cross Cancer Institute from 1 July 2014 to 31 December 2020 were retrospectively reviewed. Pts were considered eligible if they received DI for mCSPC and then received either docetaxel or cabazitaxel in mCRPC. Variables of interest were collected from electronic medical records. The primary endpoint was ≥50% PSA response at 12 weeks relative to baseline for FST. Secondary endpoints included OS from mCSPC diagnosis, as well as PFS and OS from the FST start date. PSA responses were compared using the chi-squared test, and time-based endpoints were compared using the Kaplan–Meier method. Results: In total, 34 pts were identified: docetaxel = 22 and cabazitaxel = 12 as FST. 91.2% of pts (docetaxel 95.5% vs. cabazitaxel 83.3%) received FST in 2nd line mCRPC. The median age at diagnosis (63.1 vs. 67.1 yrs, *p* = 0.236) and the median time to CRPC (18.6 vs. 14.2 mos, *p* = 0.079) were similar for docetaxel and cabazitaxel, respectively. The median time to FST (24.1 vs. 34.6 mos, *p* = 0.036) and OS from mCSPC diagnosis (30.9 vs. 52.7 mos, *p* = 0.002) were significantly shorter for pts receiving cabazitaxel vs. docetaxel. PSA responses occurred in 40.9% of pts treated with docetaxel compared to 25.0% treated with cabazitaxel (*p* = 0.645). There was no significant difference in median PFS (2.7 vs. 3.5 mos, *p* = 0.727) or median OS (11.4 vs. 8.1 mos, *p* = 0.132) from the time of FST for pts treated with docetaxel vs. cabazitaxel, respectively. Conclusions: Both docetaxel and cabazitaxel demonstrated activity as FST after DI in mCSPC. Pts who received cabazitaxel had a shorter time to FST and OS from mCSPC. The reasons for this may reflect clinician preference for cabazitaxel in pts with aggressive or rapidly progressing disease. No difference was found in PSA response, PFS, or OS from FST with docetaxel compared to cabazitaxel. While limited by its retrospective nature and small sample size, this study suggests that docetaxel is active as FST despite treatment with DI in mCSPC.

## 1. Introduction

As the prevalence of advanced prostate cancer increases (CCS), its management continues to rapidly evolve. One of the most significant changes in management over the last decade is the use of systemic therapies that were initially approved for metastatic castration-resistant prostate cancer (mCRPC) in earlier disease states, such as metastatic castration-sensitive prostate cancer (mCSPC). Since the landmark TAX-327 and SWOG 99-16 phase III trials, docetaxel has been used in mCRPC [1,2]. The benefits of the early use of docetaxel in addition to androgen deprivation therapy (ADT) in newly diagnosed patients with mCSPC were demonstrated approximately ten years later with the publication of the CHAARTED and STAMPEDE trials [3,4]. Similarly, while androgen receptor pathway inhibitors (ARPIs) such as abiraterone and enzalutamide were first approved in mCRPC, they have demonstrated significant benefits in mCSPC [3,4,5,6,7,8]. Recent evidence suggests that there may also be further benefit of combination strategies with both abiraterone and darolutamide demonstrating improved survival when added to ADT and docetaxel [6,7,9,10]. Additionally, the number of systemic therapy options for patients is significantly expanding, with several novel agents, such as PARP inhibitors and radioligand therapies, receiving regulatory approval in mCRPC and being actively investigated in mCSPC [11]. Such addition of further therapies along with ADT in mCSPC, such as ARPIs and/or chemotherapy, is referred to as treatment intensification. This approach has shown gradual uptake and is now considered the standard of care for most patients with mCSPC due to the demonstrated improvements in overall survival and time to progression [12].

Despite these advances, cytotoxic chemotherapy remains a relevant option for patients. Though approved before many of the currently available therapies, both docetaxel and cabazitaxel have demonstrated improved OS in modern clinical trials [3,5,13]. It has been demonstrated that cabazitaxel does not improve OS when used in place of docetaxel but does when used sequentially after progression on docetaxel when both are used in mCRPC [13,14]. However, the optimal sequence has not been established for when docetaxel is used initially in the mCSPC setting [15], where we are likely to see increased use given recent data. In a post-treatment analysis of patients participating in GETUG-AFU 15, only 14% of patients who received docetaxel intensification (DI) in mCSPC had a ≥50% PSA response to the subsequent use of docetaxel in mCRPC [16]. The efficacy of docetaxel relative to cabazitaxel after DI has not been demonstrated. It is important to demonstrate the outcomes of docetaxel and cabazitaxel in mCRPC after DI in mCSPC to ensure efficacy and tolerance are demonstrated when re-challenging with a taxane in this setting, as well as to guide treatment sequencing efforts for mCRPC therapies to apply to individual patients.

In this study, we aim to characterize the activity of docetaxel and cabazitaxel in mCRPC patients who have previously received docetaxel in mCSPC. By comparing the outcomes of patients who receive these agents as their first subsequent taxane (FST) after DI in mCSPC, we hope to add to the existing literature and inform treatment selection for patients and clinicians.

## 2. Methods

### 2.1. Patient Population

This study was approved by the Health Research Ethics Board of Alberta. Following approval, all new patient consults seen by the genitourinary medical oncology group at the Cross Cancer Institute, Edmonton, Canada, from 1 July 2014 to 31 December 2020 were reviewed to identify eligible patients. Patients were considered eligible for this study if they (1) received DI for mCSPC and (2) later received either docetaxel or cabazitaxel for mCRPC, irrespective of the line of therapy.

### 2.2. Data Collection Outcome Measures

Clinical data were retrieved from the regional electronic medical record (EMR). Variables of interest, including patient characteristics, treatment regimens, laboratory and imaging results, and survival outcomes, were collected from the EMR. Data were aggregated and anonymized for statistical analysis. The primary endpoint was the proportion of patients achieving ≥50% PSA response at 12 weeks relative to the baseline for FST. Secondary endpoints included OS from mCSPC diagnosis, as well as PFS and OS from the FST start date. PFS was defined as either PSA progression using Prostate Cancer Working Group criteria 3 (Scher), radiographic progression using RECIST v1.1 for soft tissue disease (Eisenhauer) and PCWG3 criteria for bone or death from any cause.

### 2.3. Statistical Analysis

Descriptive statistics were reported for the study variables. Mean and SD or median (range) were reported for continuous variables; frequencies and proportions were reported for categorical variables. PSA responses between the two groups were compared using chi-squared tests. Mann–Whitney U tests were used for median comparison between the two groups. Time-based endpoints were compared using the Kaplan–Meier method, and the survival curves were compared using log-rank tests. Univariate analysis was used to determine the factors associated with PSA response to FST. For the statistical analysis, a *p*-value < 0.05 was used for statistical significance. All statistical analyses were conducted using SPSS software version 29 (IBM Corp. Released 2022. IBM SPSS Statistics for Windows. Version 29.0. Armonk, NY, USA: IBM Corp.).

## 3. Results

Thirty-four patients (cabazitaxel = twelve; docetaxel = twenty-two) were identified, with their baseline characteristics shown in Table 1. Almost all patients (97%) received ADT and docetaxel as initial systemic therapy in mCSPC, while one patient who later received cabazitaxel as FST received an ARPI in addition to ADT and docetaxel. Nearly all patients (91%: cabazitaxel = 92%; docetaxel = 91%) received six total cycles of docetaxel in mCSPC. The median age at mCSPC diagnosis for patients was 67.1 years for cabazitaxel and 63.1 years for docetaxel (*p* = 0.236). Most patients (cabazitaxel = 100%; docetaxel = 81.8%) had bone metastases at diagnosis, with a minority having lymph node only (cabazitaxel = 0%; docetaxel = 4.5%) or visceral metastases (cabazitaxel = 0%; docetaxel =13.6%) at diagnosis with no significant difference between groups (*p* = 0.215). Baseline PSA, LDH, ALP, and neutrophil-to-lymphocyte ratio before FST were not significantly different between groups (Table 1).

At the time of the last follow-up, almost all (total 97.1%: cabazitaxel = 100%; docetaxel = 95.5%; *p* = 0.453) had disease progression on FST therapy. One patient who had received docetaxel as FST continued to be progression-free and alive at the last follow-up. Further, most patients had died at the time of the last follow-up (88.2%: cabazitaxel = 100%; docetaxel = 81.8%, *p* = 0.116).

The median time to CRPC was 17.6 months (cabazitaxel = 14.2 mos; docetaxel = 18.6 mos, *p* = 0.079). Most patients (91.2%, cabazitaxel = 83.3%; docetaxel = 95.5%) received their FST as second-line treatment for mCRPC, with few patients receiving their FST as first (5.9%: cabazitaxel = 8.3%; docetaxel = 4.6%) or third (2.9%: cabazitaxel = 8.3%; docetaxel = 0%) treatment lines in mCRPC. There was no significant difference in the line of therapy in which the FST was received between groups (*p* = 0.680).

As shown in Table 2, the median time to FST from the date of mCSPC diagnosis was significantly shorter for patients receiving cabazitaxel as FST (cabazitaxel = 24.1 mos; docetaxel = 34.6 mos; *p* = 0.036). Similarly, the time from the last docetaxel in mCSPC to FST was significantly shorter for patients receiving cabazitaxel between groups (cabazitaxel = 18.9 mos; docetaxel = 29.8 mos; *p* = 0.041). Of those who received the FST as second- or third-line therapies in mCRPC, all patients received an androgen receptor pathway inhibitor (ARPI) (28% abiraterone; 72% enzalutamide) as first-line therapy.

PSA responses, identified as ≥50% PSA reductions, at 12 weeks after the baseline from FST occurred in 35.3%, with no significant difference between groups (cabazitaxel = 25.0%; docetaxel = 40.9%; *p* = 0.465). The median PSA change at 12 weeks relative to the FST increased by 19.8% (cabazitaxel = 3.0%; docetaxel = 19.8%; *p* = 0.490) due to several patients having drastic biochemical progression since the initiation of FST. There was no significant difference between groups for PSA response as a continuous variable to FST (*p* = 0.645).

Univariate analysis was conducted to determine if any factors were associated with PSA response to FST. There was no association between time to FST (*p* = 0.294) and time from the last docetaxel in mCSPC to FST (*p* = 0.220) and response. Baseline ALP before FST, though nearing significance, was not associated with response (*p* = 0.057). Baseline PSA (*p* = 0.164), LDH (*p* = 0.606), and baseline neutrophil-to-lymphocyte ratio (*p* = 0.229) before FST were not significantly associated with response. As no factors were significant in the univariate analysis, a multivariate analysis was not conducted.

**Figure 1:**
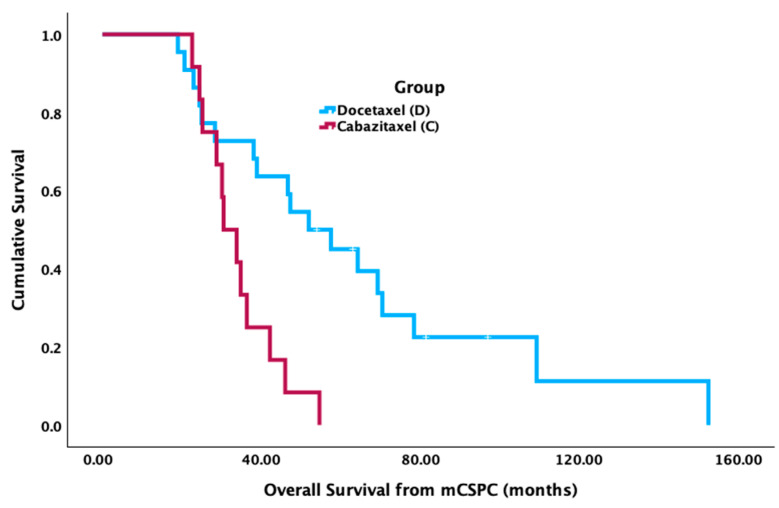
Median overall survival (OS) from metastatic castrate-sensitive prostate cancer (mCSPC) diagnosis between cabazitaxel and docetaxel.

**Figure 2:**
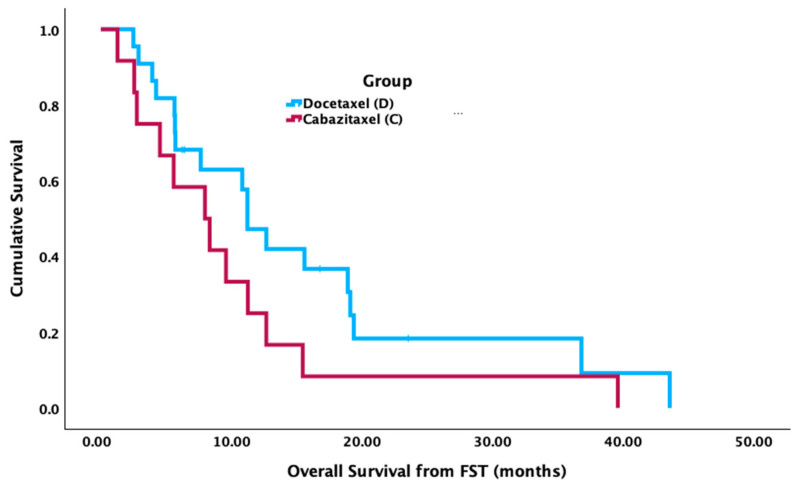
Median overall survival (OS) from first subsequent taxane (FST) start date between cabazitaxel and docetaxel.

**Figure 3:**
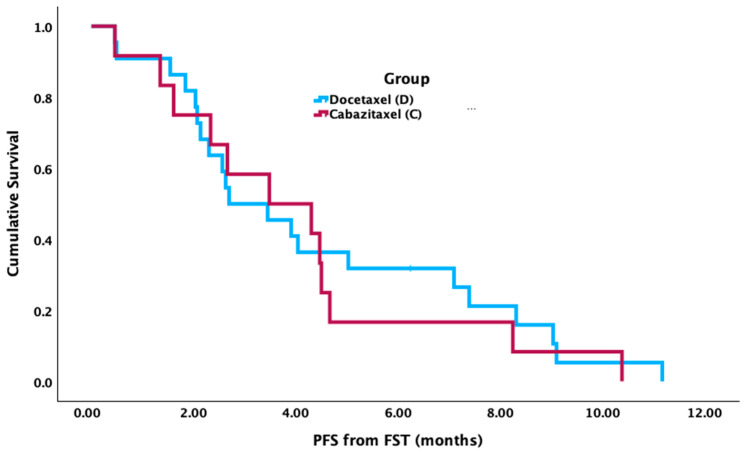
Median progression-free survival (PFS) from first subsequent taxane (FST) start date between cabazitaxel and docetaxel.

Patients who received docetaxel in FST had significantly longer median OS from mCSPC diagnosis (52.7 mos [95% CI 36.4–69.0 mos]) compared to cabazitaxel (31.0 mos [95% CI 24.6–37.3 mos] (*p* = 0.002) (Figure 1). There was no significant difference in median OS from the FST start date between groups (cabazitaxel = 8.1 mos [95% CI 3.3–12.9 mos]; docetaxel = 11.4 mos [95% CI 8.8–14.0 mos], *p* = 0.132) (Figure 2). There was no significant difference between groups for median PFS from the FST start date (cabazitaxel = 3.5 mos [95% CI 0.7–6.4 mos]; docetaxel = 2.7 mos [95% CI 1.2–4.3 mos], *p* = 0.727) (Figure 3).

## 4. Discussion

This study found that both docetaxel and cabazitaxel were active in mCRPC after DI in mCSPC, with PSA response rates comparable to the registration trials for these agents that were conducted in mCRPC patients who did not receive DI [1,13]. No significant difference in response rates was seen between docetaxel and cabazitaxel, nor were differences in PFS or OS from the start of FST. Specifically, in this study, docetaxel showed a PSA ≥50% response rate of 40.9%, which is notably higher than what was reported by Lavaud et al. [16], where a PSA ≥ 50% response was seen in only four of twenty-nine patients (14%). There did not appear to be a clear advantage of using cabazitaxel as FST after DI, which showed a numerically lower response rate compared to docetaxel. Baseline characteristics were similar between patients who received docetaxel or cabazitaxel, including known prognostic factors. Baseline ALP, LDH, neutrophil/lymphocyte ratio (NLR), PSA, total PSA response, and the presence of visceral metastases have previously been identified as important prognostic factors in mCRPC patients [17,18,19,20,21,22,23]. In this study, baseline ALP (*p* = 0.057), baseline PSA (*p* = 0.164), LDH (*p* = 0.606), and NLR (*p* = 0.229) before FST were not predictive of biochemical treatment response but were not analyzed prognostically.

Notably, the time to FST and time from the last docetaxel was significantly shorter for patients receiving cabazitaxel compared to docetaxel. This may reflect clinicians’ perception that cabazitaxel is more active after docetaxel and a preference to select this in patients they believe to have more aggressive disease due to the relatively rapid development of mCRPC. The patients in the cabazitaxel group had a worse prognosis, as reflected in a worse OS from mCSPC diagnosis. This, however, did appear to not impact outcomes with FST after DI with no baseline factor, including time to mCRPC or time from the last docetaxel, associated with probability of response on univariate analysis. Similarly, there was no significant difference in PFS or OS from FST, which would suggest the differences in OS from mCSPC were related to prognostic factors rather than their outcome with FST therapy. The *p*-value, however, was low (0.132), which may indicate a trend toward differences in overall survival from FST between docetaxel and cabazitaxel, which would be better assessed through a larger sample size.

Upon a review of the literature, FIRSTANA is the only study to directly compare the efficacy of cabazitaxel and docetaxel in mCRPC patients; however, this study used a chemotherapy-naïve population. FIRSTANA found no significant difference in OS between groups [14]. The findings of this study are consistent with this research, suggesting that the prior use of DI in mCSPC does not influence the outcome of taxane chemotherapy in mCRPC. These findings could be used to guide patient-specific taxane selection, acknowledging a patient preference for cabazitaxel with enhanced quality of life and reduced fatigue, pain, nausea/vomiting, and hair/nail changes when compared to docetaxel with reduced diarrhea [14,24].

This study is limited by its retrospective nature and small sample size, as patients had to receive taxane chemotherapy in mCSPC and mCRPC to meet eligibility. Another possible limitation is its lack of standardization of drug sequencing, where patients received cabazitaxel and docetaxel in various treatment lines after different systemic therapies. Further studies are needed to determine the best sequencing of and comparisons between survival-prolonging systemic therapies in mCRPC. Additionally, further studies are needed to determine outcomes following the use of FST in mCRPC after DI in mCSPC. There is evidence for the re-challenge of taxanes, specifically docetaxel, in mCRPC, but little is known regarding the use of taxanes in mCRPC after DI in mCSPC due to the timing of initial studies.

## 5. Conclusions

This study compared the use of taxane chemotherapies, specifically docetaxel and cabazitaxel, in mCRPC following DI in mCSPC. Its findings contribute to patient-specific treatment selection and sequencing efforts in mCRPC. Both docetaxel and cabazitaxel demonstrated activity as FST after DI in mCSPC. Patients who received cabazitaxel had a significantly shorter time to FST and a shorter median OS from mCSPC. No difference was found between docetaxel and cabazitaxel for PSA response, PFS, or OS from the FST. While limited by its retrospective nature and small sample size, this study suggests that docetaxel and cabazitaxel are active as FST after treatment with DI in mCSPC. Further research, such as a multicentre analysis, is needed to compare docetaxel and cabazitaxel as FST in mCRPC after DI in mCSPC. 

## Figures and Tables

**Table 1:** Baseline patient characteristics.

	Cabazitaxel	Docetaxel	*p*-Value
Total Patients	12	22	
Median age at mCSPC diagnosis (years)	67.1	63.1	0.236
Received an ARPI in mCSPC	8.3%	0%	
Sites of metastases			0.215
Bone (+/− lymph node)	100%	81.80%	
Lymph nodes only	0%	4.50%	
Visceral	0%	13.60%	
Baseline laboratory values before FST			
PSA	128.1	24.5	0.093
LDH	178.0	187.0	0.148
ALP	261.0	246.0	0.260
Neutrophil-to-lymphocyte ratio	3.3	2.6	0.374
Progression at last follow-up	100%	95.50%	0.453
Surviving at last follow-up	0%	18.20%	0.116
Median time to CRPC (months)	14.2	18.6	0.079

**Table 2:** Treatment and survival outcomes.

	Cabazitaxel	Docetaxel	*p*-Value
Median time to FST (months)	24.1	34.6	0.036
Time from last docetaxel in mCSPC to FST (months)	18.9	29.8	0.041
Received FST as second-line	83.30%	95.50%	
PSA ≥ 50% response to FST	25.00%	40.90%	0.645
Median OS from mCSPC diagnosis (months)	31	52.7	0.002
Median OS from FST start date (months)	8.1	11.4	0.132
Median PFS from FST start date (months)	3.5	2.7	0.727

## Data Availability

The data presented in this study are not available due to privacy or ethical restrictions.
